# Impacts of Thermal and Mechanical Cycles on Electro-Thermal Anti-Icing System of CFRP Laminates Embedding Sprayable Metal Film

**DOI:** 10.3390/ma14071589

**Published:** 2021-03-24

**Authors:** Rongjia Li, Wang Xu, Dalin Zhang

**Affiliations:** Institute of Man-Machine & Environment Engineering, College of Aerospace Engineering, Nanjing University of Aeronautics and Astronautics, Nanjing 210016, China; Rongjia_Li_NUAA@outlook.com (R.L.); xuwang_nuaa@outlook.com (W.X.)

**Keywords:** CFRP composite, sprayable metal film, cyclic loading, functional fatigue, anti-icing system

## Abstract

The aircraft electro-thermal anti-icing system that can guarantee flight safety may be affected by periodic heating and cyclic aerodynamic force during long-term flight missions, which seems to be a potential threat to ice protection. This paper aims to investigate the impacts of thermal and mechanical cycles on heating elements of the electro-thermal anti-icing system. Specimens were manufactured with CFRP (carbon fiber reinforced polymer) laminated composite, glass fiber prepreg and copper screen, in which sprayable metal film (SMF) was embedded as the heating element. The study focuses on electric resistance variation of SMF and functional fatigue life under the cycling load. Thermal cycling tests were carried out in an insulated chamber where the specimens were heated up to 80 °C and then cooled down to −55 °C for 1000 cycles. Mechanical cycling tests were conducted on a fatigue testing machine where the specimens were imposed on tension-compression loading for 10^6^ cycles. Results showed that the electric resistance of SMF increased with the number of loading cycles. The resistance was increased by 20% and the heating power was decreased by 16.67% after 1000 thermal cycles. During the mechanical cycling tests, it was found that the heating element was destructed before the structural failure, which indicated that the fatigue life of function was lower than that of the structure.

## 1. Introduction

When an aircraft is flying through clouds with super-cooled water droplets, it has a great chance of icing. The icing on aircraft wings and stabilizers can result in drastic degradation of aerodynamic and maneuvering performances, which threatens flight safety [1]. To mitigate the detrimental impacts of ice accretion, many ice protection systems have been developed, such as hot-air and electro-thermal anti-icing systems [2].

Recently, the application of composite materials on aircraft increased significantly due to their remarkable properties such as high strength-to-weight ratios, corrosion resistance, and excellent fatigue endurance. For instance, carbon fiber reinforced polymer (CFRP) composites have been the predominant materials on the primary structure of Airbus A350 XWB [3] and Boeing 787 [4]. However, CFRP composite usually has poor thermal conductivity. The hot-air anti-icing system with CFRP composite requires a large amount of hot air bled from engines, which reduces the efficiency of the engines and adds weight as well as maintenance. The electro-thermal anti-icing system with CFRP composite has the advantages of simple and clean installation, convenient controllability, and efficient thermal response [5], which has attracted considerable research interest. This combination becomes highly applicable to the development of future aircraft that demands lightweight and eco-efficient systems and structures [6].

CFRP composites are commonly employed as the main structure of the electro-thermal anti-icing system. To improve system integration, the heating elements are usually embedded in CFRP laminates. In recent years, a variety of materials were developed as heating elements, including metal materials, graphene [7,8], carbon fibers [9], carbon nanotubes (CNTs) [10,11,12,13], and electro-conductive textile [14]. Among these, metal materials are widely used due to their excellent stability, reliability, and operability. Boeing 787 makes use of metal spraying technology to deposit the heating element onto a glass fiber fabric [15]. Mohseni [16] developed the electro-thermal anti-icing system in the form of discrete constantan wires with a specific pattern inside the composite airfoil to protect wind turbine from icing. Carbon-based materials are potential alternatives as they have light weight and nice compatibility with CFRP matrix. Chen [17] adopted a graphene coating to improve the efficiency of the anti-/de-icing system for a helicopter blade. Lu [18] developed a flexible and transparent thin-film heater composed of carbon fibers and regenerated cellulose, which exhibited uniform heating and rapid thermal response. Buschhorn [19] used Joule heating of aligned CNT arrays to create highly efficient de-icing and anti-icing of aerosurfaces.

When the heating element is in a working state, its temperature will be raised, which imposes thermal loading on the electro-thermal anti-icing system. Besides, the system mounted on aerodynamic components such as wings and stabilizers will suffer a variety of mechanical loading due to lift and drag during the flight. After several flying assignments, the influences of the thermal and mechanical cycling loading will be accumulated, which may lead to adverse effects on the structure and performance of the system. Alderliesten [20] developed methods to study the fatigue and damage tolerance of GLARE (glass fiber reinforced aluminum) [21] which was used in the wing structure of the Airbus A380. Müller [22] designed a thermal cycling setup to investigate the effects of thermal cycling on the material properties of GLARE and found out the interlaminar shear strength increased with the number of cycles. Hagenbeek [23] conducted experiments of long-term thermal cycling and moisture on heated fiber metal laminates and glass-fiber epoxy composites to research the durability of structure. Subsequently, Hagenbeek [24] studied the effects of thermal cycling on heated fiber metal laminates under static load. The results showed that the addition of a static load has a distinct influence on the interlaminar shear properties after thermal cycling. Gigliotti [25] employed the electrical resistance measurements to assess damage onset and development within the composite structure and focused on the electro-mechanical fatigue of CFRP laminates. Harizi [26] used the electrical resistance measurements during self-heating tests on continuous carbon fiber polymer-matrix composites for rapid estimation of their strength fatigue limit. Khosravani [27] presented an experimental investigation on the effect of accelerated ageing on the mechanical properties of perpendicular honeycomb sandwich connections. Fujimoto [28] investigated the low cycle fatigue of CFRP laminated composites due to repeated out-of-plane loading by four-point-bending fatigue test and flatwise tension fatigue test.

In addition, some numerical research related to laminates structure is also devoted to understanding the fatigue process. Funari [29] developed a new methodology based on a moving mesh technique and a multilayer formulation to predict crack onset, evolution and coalescence of the interlaminar damage mechanisms. Xu [30] enhanced the conventional extended finite element method to simulate dynamic crack branching. Hwang [31] studied the fatigue behavior of glass fiber reinforced epoxy materials and predicted the fatigue life using the fatigue modulus and its degradation rate. Vassilopoulos [32] adopted an artificial neural network to study the fatigue life of multidirectional composite laminates made of GFRP composite materials. Lian [33] simulated the fatigue damage evolution in composite laminates and predict fatigue life of the laminates with different lay-up sequences on the basis of the fatigue characteristics of longitudinal, transverse and in-plane shear directions by finite element analysis (FEA) method.

Much research has covered the behaviors of composite structure under a series of complex thermal and mechanical cycles in terms of strength, integrity and stiffness. However, the operating circumstances of electro-thermal anti-icing constructed with CFRP laminates under cyclic load is affected not only by the mechanical properties of structures, but also by the characteristics of heating elements, such as the electric resistance variation and the output heating power. Additionally, if the functional life expectancy is not predicted correctly, the anti-icing system may be defunct in flight, leading to the risk of ice accretion. Consequently, it is necessary to study the system functional lifetime under thermal and mechanical cycles.

To the author’s knowledge, the impacts of cyclic load on the functional fatigue life of electro-thermal anti-icing system have not been reported before. In this paper, a detailed study about the effects of thermal and mechanical cycles on the electro-thermal anti-icing system comprising CFRP laminated composite in which sprayable metal film (SMF) was embedded as the heating element was presented, aiming at revealing the resistance characteristics of SMF and the functional fatigue life of the system. Different specimens were manufactured according to the structure of the system and experiments including temperature test, static tensile test, thermal and mechanical cycling tests were carried out. The electric resistance variation of SMF was measured along with the change of temperature, strain and the number of cycles. The functional fatigue life was obtained under different cyclic load and compared with the structural fatigue life.

The paper is organized as follows. Section 2 describes the specimen preparation and the experimental setup employed in the thermal and mechanical cycling tests. Section 3 presents the obtained results including the variation of electric resistance under different test conditions and discusses the approach to improving the functional fatigue life of electro-thermal anti-icing system. Finally, the conclusion is drawn in Section 4.

## 2. Materials and Experimental Procedures

### 2.1. Materials and Specimen Fabrication

**Figure 1:**
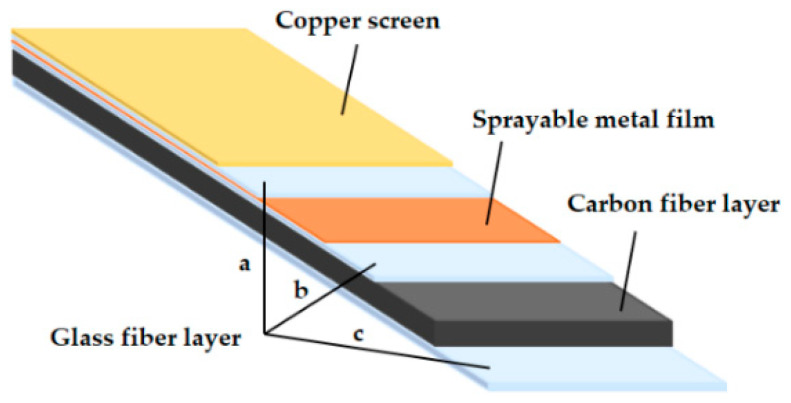
Schematic lay-up of a specimen: (a) for insulation between copper screen and sprayable metal film; (b) for insulation between sprayable metal film and carbon fiber layer; (c) for insulation between carbon fiber layer and the outside.

The specimens of the electro-thermal anti-icing system comprising CFRP composite and SMF were manufactured with copper screen, glass fiber layers, SMF, and carbon fiber layers, as displayed in Figure 1. The copper screen with the mesh count of 10 was designed to resist lightning strikes and parasite electric currents. The SMF, which was made of Cu-Mn alloy, was deposited inside the specimen as the heating element through the metal thermal spray technology by United Coatings Technologies (Beijing, China) Co., Ltd. The film which formed the alloy coating containing 79.10% Cu and 20.90% Mn, was found to be 100 µm (±5 µm) in thickness after the deposition. As can be seen from Figure 1, the SMF was close to the upper surface instead of the middle in the thickness direction, which can reduce thermal resistance and improve heat transfer efficiency in the anti-icing process. The carbon fiber layers with a stacking sequence of (45/−45/0/90/45/0/90/−45/0)_s_ were molded by autoclave processing (max curing temperature: 200 °C) to enhance the structural strength of the specimen. To improve the electrical insulation between the SMF and other layers, the glass fiber layers with a stacking sequence of (0/90) were placed between the SMF and the carbon fiber layers. The composite materials and the processing technology were provided by Aerospace Haiying (Zhenjiang, China) special materials Co., Ltd. The material specifications are listed in Table 1.

**Figure 2:**
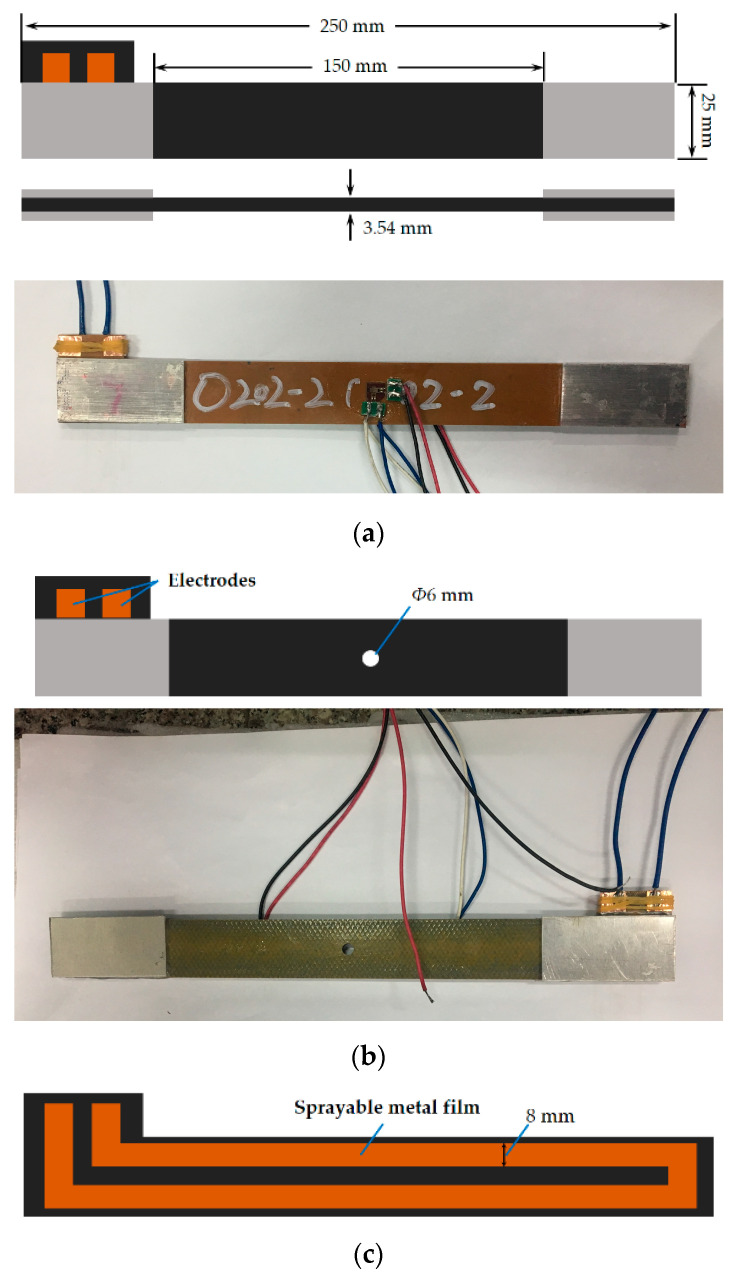
Dimensions of specimens: (**a**) specimen used in temperature and static tensile tests; (**b**) specimen used in thermal and mechanical cycles tests; (**c**) circuit of the sprayable metal film (SMF).

Figure 2 illustrates the dimensions of the specimens, which were determined by the ASTM standards [34,35]. The clamping area is at the end of the specimen and the loading area is in the middle of the specimen. The specimen in Figure 2a was used in temperature and static tensile tests, while the specimen in Figure 2b with a hole of Φ6 mm in the center was used in thermal and mechanical cycling tests. The circuit of the SMF is displayed in Figure 2c, which is extended a little as the electrodes at the edge of the specimen.

### 2.2. Experimental Tests

#### 2.2.1. Thermal Cycling Test

**Figure 3:**
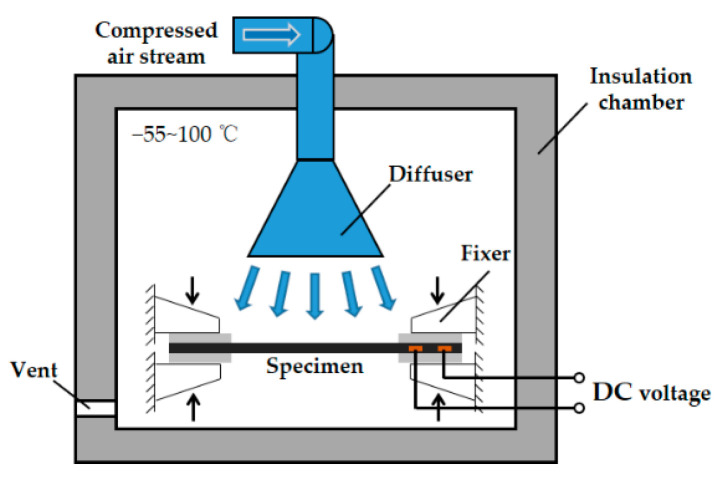
Schematic diagram for thermal cycling test.

Before the thermal cycling test, several specimens were kept in an insulation chamber to measure the variation of the electric resistance along with the temperature. The insulation chamber used the compressed air stream with different temperatures to adjust the internal temperature which can be varied from −55 to 100 °C. During the test, the specimens were clamped by the fixers inside the chamber to mitigate the influences of the temperature variation on the expansion and contraction of the specimen, as illustrated in Figure 3.

**Figure 4:**
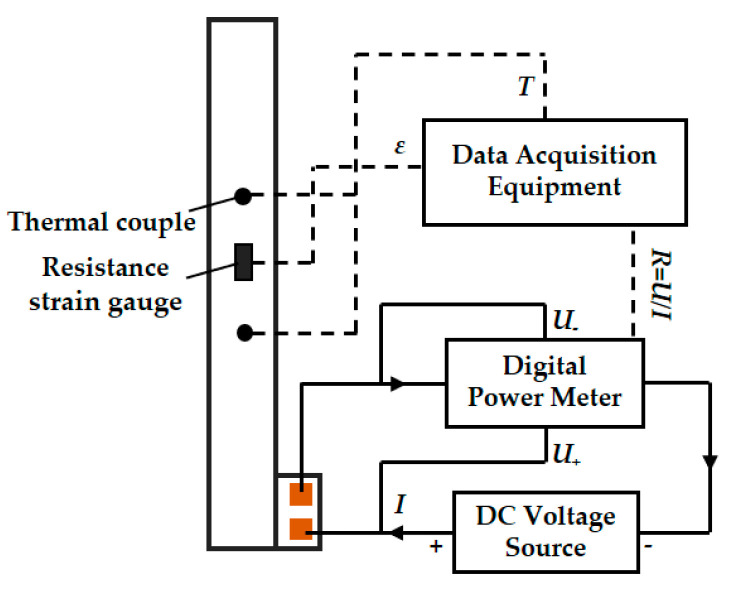
Measurements of temperature, strain and electric resistance.

**Figure 5:**
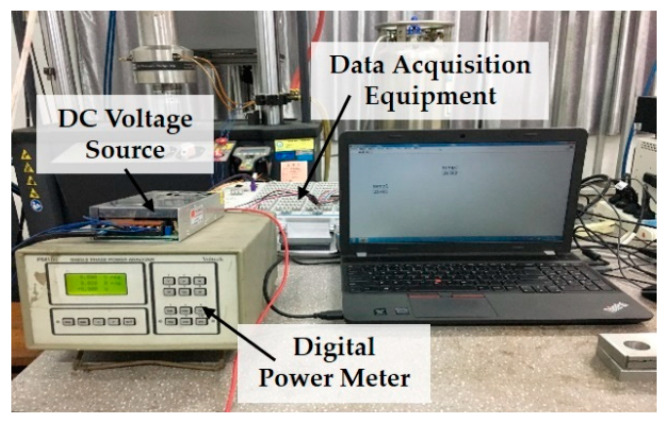
Apparatus used in the measurements.

Figure 4 shows the schematic measurements of electric resistance, strain and temperature. A DC voltage source was adopted to impose a weak voltage on the specimen and a digital power meter (PM100, Voltech, Abingdon, UK) was used to measure the voltage and current and calculate the electric resistance by Ohm Law. As the electric power was quite low, the self-heating effect caused by the current can be neglected. Two resistance strain gauges (BE120-3BC, JINGCE DIANQI, Hanzhong, China) were attached on both sides of the specimens in the center to measure the strain variation. T-type thermocouples were attached to the specimen surface to monitor the temperature. An integrated National Instrument (NI) system (National Instruments, Austin, TX, USA) was utilized as the data acquisition equipment. Figure 5 displays the apparatus used in the measurements.

The thermal cycling test was conducted with the same apparatus employed for the temperature test. During the test, temperature, strain and electric resistance of the specimens were acquired by the data acquisition equipment. It was different from the temperature test that the temperature inside the chamber was maintained at −55 °C in the thermal cycling test. In a single cycle, the specimen was heated by the DC voltage from −55 to 80 °C and cooled down to −55 °C by the cold air stream. The temperature boundary of the thermal cycle was determined by the designed operating temperature range of the electro-thermal anti icing system. When the aircraft is flying at high altitude, the system in a standby state can be affected by the ambient temperature as low as −55 °C, while the temperature of the system is limited to 80 °C in a working state according to the mechanical properties of the structure at high temperature [36,37]. The heating power density was controlled at 30 kW/m^2^. The number of thermal cycles was 1000. The change of the electric resistance with the number of thermal cycles was measured.

#### 2.2.2. Mechanical Cycling Test

**Figure 6:**
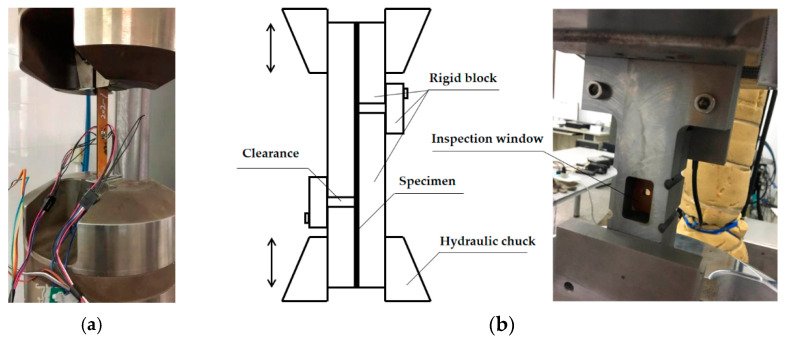
Mechanical tests apparatus: (**a**) static tensile tests; (**b**) cycling tests.

Before the mechanical cycling test, a static tensile test was conducted at a speed of 2 mm/min using an electrical tensile machine (MTS 370.25, MEASURE TEST SIMULATE, Eden Prairie, MN, USA) with a load cell of 250 kN, as displayed in Figure 6a. The temperature during the test was kept at room temperature (25 °C). The measurements of electric resistance and strain employed the apparatus used in the thermal cycling test. Note that the specimen was loaded with a weak voltage by the DC voltage source, so that the digital power meter can measure the electric resistance in real time without heating the specimen. The variation of the electric resistance Δ*R*/*R*_0_ can be defined as:

(1) $$\frac{\Delta R}{R_{0}}=\frac{R_{\varepsilon }-R_{0}}{R_{0}}$$

where *R*_0_ is the initial electric resistance and *R*_ε_ is real-time electric resistance.

The mechanical cycling test executed tension-compression cycles, which was carried out on a fatigue testing machine (MTS 370.10, MEASURE TEST SIMULATE, Eden Prairie, MN, USA). Then, 10^6^ cycles were applied with a frequency of 5 Hz and load ratio (r) of −1. Before the test, several specimens with an opening hole in the center were used to measure the ultimate strength of tension and compression, of which the results were 400 and 370 MPa, respectively. Therefore, the amplitude of the sinusoidal load spectrum applied in the mechanical cycling test was based on the percentage of the ultimate strength of compression. Table 2 exhibited the experimental conditions in the mechanical cycling test. As the specimens were subjected to tensile and compressive stress, a pair of special testing clamps were prepared, which is displayed in Figure 6b. The ends of the specimen were clamped by the hydraulic chuck, and the stiffness in the compressive process was maintained by the rigid blocks around the specimen. There was a movable gap between the rigid blocks to ensure that the tensile and compressive process was not disturbed. The middle inspection window can be used to observe the damage near the opening hole during the test.

## 3. Results and Discussion

### 3.1. Temperature Test

**Figure 7:**
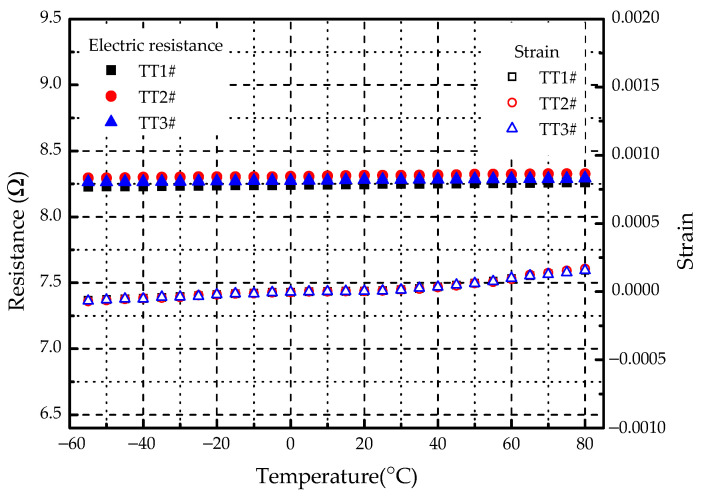
Variation of electric resistance and strain along with temperature.

**Figure 8:**
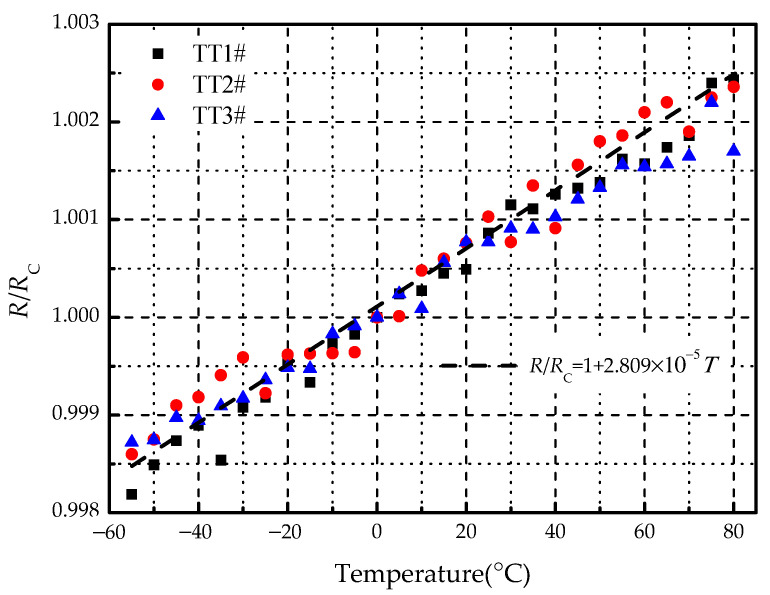
Variation of *R*/*R*_C_ along with temperature.

Before the thermal cycling test, a temperature test was carried out on three specimens (TT1#, TT2#, and TT3#), of which the results are shown in Figure 7. It can be seen that the electric resistances of three specimens had deviations due to the limitation of metal spraying level. The maximum deviation was within 1%, which was ignored in the test. The strain had little change at low temperatures due to the clamps of specimen ends. However, it started to increase with the temperature because of thermal expansion. In the temperature range of −55 to 80 °C, and the electric resistance was almost unchanged. It is well known that the relationship between the temperature and the electric resistance can be formulated as [38]:

(2) $$R=R_{C}\left(1+\alpha T\right)$$

where *R*_C_ is the electric resistance at 0 °C, *α* is the temperature coefficient of resistance (TCR) and *T* is the temperature. Based on the electric resistance at 0 °C, the results of *R*/*R*_C_ with temperature are displayed in Figure 8. By linear regression fitting, the relationship between *R*/*R*_C_ and *T* can be obtained. The TCR of SMF can be derived from the slope of the linear relationship. The calculated TCR is 2.809 × 10^−5^ (1/°C) and the standard deviation is 6.595 × 10^−7^. The range of the standard TCR provided by the manufacturer is 2.8~3.0 × 10^−5^ (1/°C). It means that the experimental results are acceptable. Considering that the temperature of the specimen was not uniform, the local temperature measured by the thermocouple was regarded as the average temperature of the specimen, which may result in errors.

Noted that the TCR of SMF has a positive value, indicating that the electric resistance increases with the rise of temperature. Supposed that the maximum operating temperature range of the electro-thermal anti-icing system is from −55 to 80 °C, the variation of electric resistance varies between −0.21% and +0.17% based on the ground temperature of 20 °C. This means that the change of temperature has little influence on the electric resistance of SMF.

### 3.2. Thermal Cycling Test

**Figure 9:**
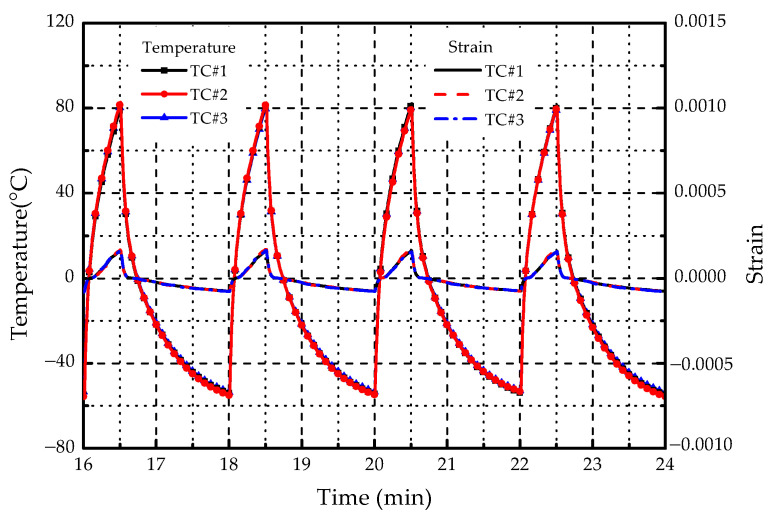
Variation of temperature and strain in thermal cycles.

**Figure 10:**
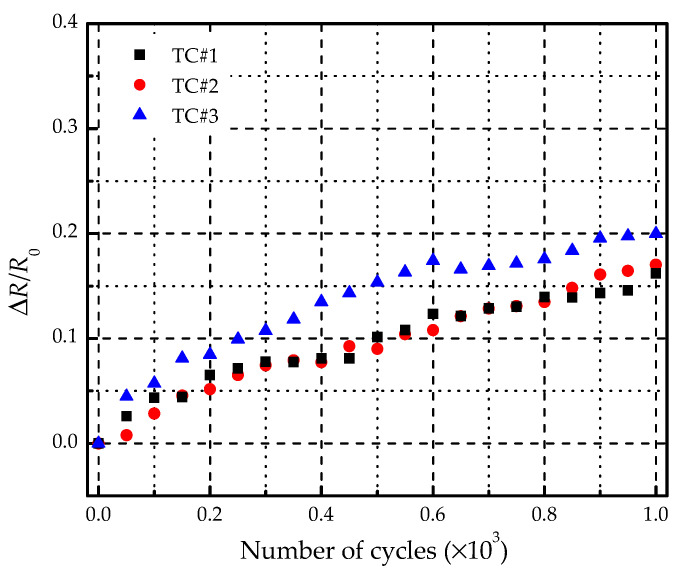
Variation of electric resistance along with the number of thermal cycles.

Figure 9 displays the change of temperature and strain in four thermal cycles. The resulting time per thermal cycle was about 2 min. The temperature of the specimen increased from −55 to 80 °C, and then dropped to −55 °C. Although the strain did not change much in thermal cycles, it still had a large value as the temperature reaches 80 °C, which means that the specimen had a slight deformation during the cycling test. Figure 10 shows the electric resistance variation along with the number of thermal cycles. The initial resistances of TC1#, TC2#, and TC3# were 8.29, 8.36 and 8.23 Ω, respectively. As the number of cycles increased, the electric resistance tended to be raised. After 1000 thermal cycles, the resistances became 9.58, 9.73, and 9.94 Ω. The maximum increment was about 20%. Supposed that the supply voltage of the electro-thermal anti-icing system remains constant, the heating power *Q* can be calculated according to:

(3) $$Q=\frac{U^{2}}{R}$$

where *U* is the loading voltage and *R* is the electric resistance. As the resistance is increased by 20%, the heating power is decreased by 16.67%, which degrades the performance of the electro-thermal anti-icing system. No visually detectable defects were found after thermal cycles in the specimens. Furthermore, the ultrasonic C-scan results showed that there was neither delamination nor pores before and after thermal cycles. It indicated that the deterioration of heating performance preceded the destruction of structure.

### 3.3. Static Tensile Test

**Figure 11:**
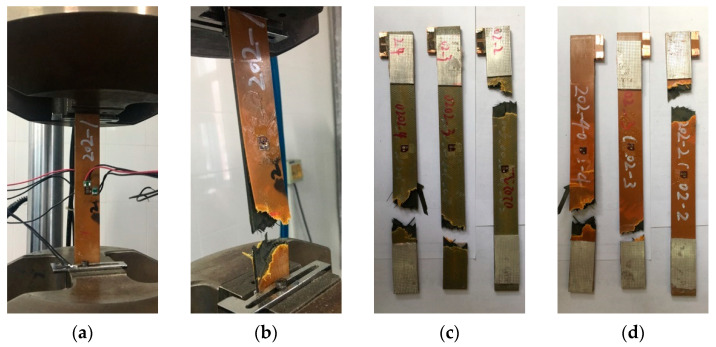
Specimens used in the measurement of mechanical properties: (**a**) specimen in the test; (**b**) specimen after the test; (**c**) front of specimen; (**d**) back of specimen.

Before the mechanical cycling test, a static tensile test was conducted. Firstly, several specimens were used to measure the mechanical properties, as displayed in Figure 11. The average tensile elastic modulus of the specimen was 40.6 GPa and the average ultimate strength was 825.4 MPa.

**Figure 12:**
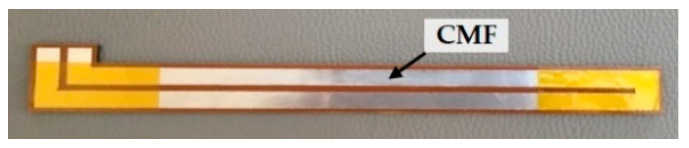
Specimen with conventional metal film (CMF) affixed on the surface.

**Figure 13:**
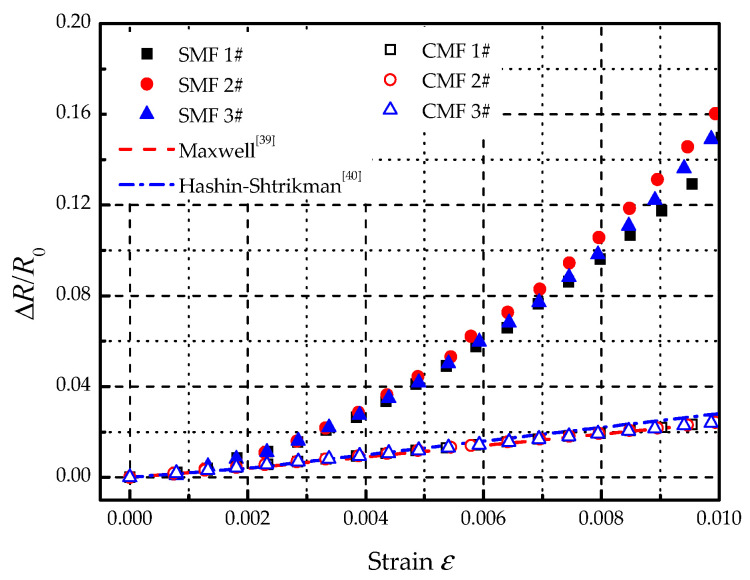
Variation of electric resistance along with strain.

Then, to compare the electric resistance variation characteristics between SMF and conventional metal film (CMF), a metal film of Ni-Cr alloy material was affixed on the surface of a specimen, as illustrated in Figure 12. The strain of specimen during the test was controlled within 0.01, of which the stress did not exceed 50% of ultimate strength. The maximum increment of the electric resistance of SMF was about 16%, and the output heating power was decreased by 13.8% according to Equation (3). The results are shown in Figure 13. As can be seen that the electric resistances of both SMF and CMF increased with the strain. For CMF, there have been equivalence theories that can describe the variation, such as Maxwell [39] and Hashin-Shtrikman [40]. As displayed in Figure 13, comparing with the experimental results of CMF, the average deviations were within 3% and 10% for Maxwell and Hashin-Shtrikman respectively, which showed a good consistency. The experimental results of SMF were close to the equivalence theories when the strain was small. However, the deviation became larger and larger as the strain increased. It indicates that the equivalence theories for CMF are not suitable for SMF.

**Figure 14:**
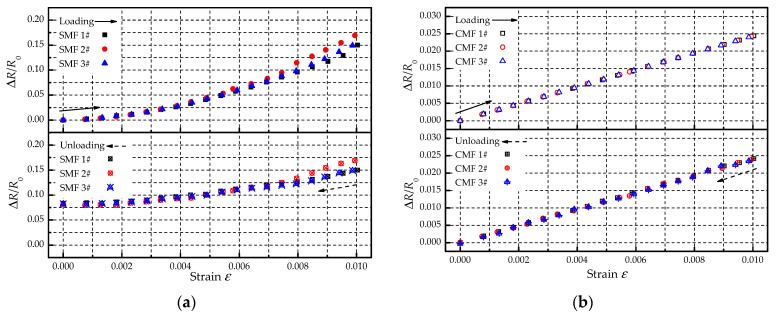
Variation of electric resistance during the loading-unloading process: (**a**) SMF; (**b**) CMF.

After being subjected to a 50% ultimate tensile strength load, the specimens were slowly unloaded. The variations of electric resistances of SMF and CMF during the unloading process are shown in Figure 14. As can be seen that the electric resistances of both SMF and CMF decreased with the strain reduction. When the stain reached zero, the electric resistance of CMF was almost the same as the initial value. However, the electric resistance of SMF was about 8% higher than the initial value. The results indicated that CMF and SMF showed different electric resistance characteristics after loading and unloading. CMF is usually a continuous medium with a compact microstructure, which has an elastic deformation stage before the crack. Therefore, the material will not be affected by loading and unloading. SMF is formed by spraying and depositing metal particles, and its microstructure often has pores. When subjected to tensile load, the scale of pores will continue to expand, and the number of pores will gradually increase. These irreversible effects will increase the resistivity of the material, resulting in a permanent increase in the electric resistance of SMF after loading and unloading.

### 3.4. Mechanical Cycling Test

The results of the mechanical cycling test are listed in Table 3. The number of cycles when the structure of the specimen is damaged is defined as the structural fatigue life, and the loading cycles when the electric current is zero is defined as the functional fatigue life. The results of F1 and F2 reached the target cycles, and no obvious cracks were found on the surface. The electric resistances of F1 and F2 were increased by 10.2% and 8.9%. The results of F3 and F4 failed to reach 50% of the target cycles. The circuit of SMF was damaged before the structure fracture. The specimens F5 and F6 were destroyed within 20,000 loading cycles, and their structure and function failed simultaneously.

**Figure 15:**
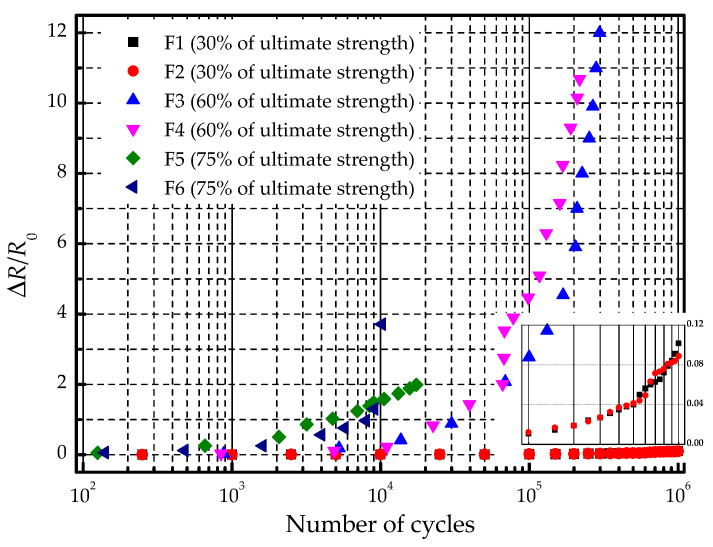
Variation of electric resistance along with the number of mechanical cycles.

Figure 15 shows the variation of electric resistance along with the number of mechanical cycles. Under 30% of ultimate compressive strength, the electric resistance values of F1 and F2 increased slowly with the loading cycles. Under 60% of ultimate compressive strength, as the cycle reached about 30,000, the resistance value was increased by 100%. As a consequence, the output heating power became 50% of the initial value, which will seriously degrade the performance of the electro-thermal anti-icing system. What is more, the resistance value was doubled when the number of cycles is less than 6000 under 75% strength. Obviously, when the amplitude of the mechanical cycles is low, the increment of the electric resistance is small, and the specimen has a long functional fatigue life. However, when the amplitude of the mechanical cycles becomes higher, the electric resistance will be increased by 100% after short cycles and the heating element becomes defunct very soon before the structural damage of the specimen. This indicates that the functional fatigue life of the specimen is shorter than the structural fatigue life under the mechanical cycling loading.

Although the electric resistance of SMF will increase and the output heating power will decrease after thermal and mechanical cycles, the following suggestions may mitigate the problem and improve the functional fatigue life:

- The structure layer can be constructed with materials of higher elastic modulus, and the stacking sequence of carbon fiber can be optimized to improve the stiffness. In the case of a certain load, the greater the stiffness of the structure, the smaller the deformation. The electric resistance of SMF will increase considerably when the strain has a large value due to their nonlinear relationship. If the strain can be controlled at a small value during the flight, the increase of the electric resistance can be effectively restrained.
- The manufacturing process of SMF can be improved to reduce the porosity and densify the deposition, so as to make SMF closer to the continuous medium. If SMF has an elastic deformation stage, it can return to or approximate the initial state after a certain load and the functional fatigue life can be improved.

## 4. Conclusions

In this paper, the impacts of thermal and mechanical cycles on electro-thermal anti-icing system were investigated. Specimens of CFRP laminates embedded with SMF as the heating element were tested under thermal and mechanical cycles. The real-time electric resistance of SMF along with the number of cycles was measured to find out the influence of cycling loads on the heating performance and fatigue life of the system. The results are summarized as follows.

- The TCR of SMF measured in the temperature test has a positive value, which indicates that the electric resistance increases with the rise of temperature. Nevertheless, within the system operating temperature range of −55~80 °C, the change of the electric resistance is no more than ±0.2%. It can be considered that the temperature change has little influence on the electric resistance of SMF. In the thermal cycling test, it is found that the electric resistance gains with the number of cycles. After 1000 cycles, the electric resistance was increased by 20% and the output heating power was decreased by 16.67%. The thermal cycle will lead to a heating performance degradation without affecting the structural integrity of the system.
- In the static tensile test, it is found that the electric resistance of SMF increases nonlinearly with the strain. After the loading-unloading process, the electric resistance of SMF becomes larger than that of initial state. Under the repeated mechanical cycles, this effect will be accumulated and strengthened, which will give rise to the increase of the electric resistance with the number of cycles. Additionally, the various amplitude of the mechanical cycle has a different influence on the fatigue life of the specimen. The larger the amplitude of the mechanical cycle, the shorter the fatigue life of the specimen. Before the structural damage of the specimen, the output heating power starts to decrease and the heating performance is degraded, which indicates that the functional fatigue life is shorter than the structural fatigue life.

The provided results of the present study may help to predict the functional fatigue life of electro-thermal anti-icing system with the structure of CFRP laminates embedding SMF and guide the margin design of heating performance on ice protection. The problem of the system behavior under a complex combination of thermal and mechanical cycles is the further study, which needs to be carried out in the near future.

## Figures and Tables

**Table 1:** Material specification.

Material		Thickness, mm	Designation
Copper screen		0.3	CMS-AD-107-II-2-B-022
Glass fiber layers	a	0.2	CMS-CP-313-I-4-120
	b	0.2	
	c	0.4	
SMF		0.1	Cu-Mn alloy
Carbon fiber layers		2.34	CMS-CP-306-35-1-130

**Table 2:** Specimens in mechanical cycling test.

Specimens	Stress, MPa	Frequency, Hz	Target Cycles	Remark
F1	111	5	1,000,000	30% of compressive ultimate strength
F2				
F3	222	5	1,000,000	60% of compressive ultimate strength
F4				
F5	277.5	5	1,000,000	75% of compressive ultimate strength
F6				

**Table 3:** Results of mechanical cycling test.

Specimens	Initial Resistance, Ω	Final Resistance, Ω	Structural Fatigue Life (Cycles)	Functional Fatigue Life (Cycles)
F1	13.09	14.42	1,000,000	1,000,000
F2	12.39	13.49	1,000,000	1,000,000
F3	11.78	∞	422,591	296,698
F4	11.65	∞	461,653	217,502
F5	10.06	∞	17,370	17,370
F6	12.75	∞	10,686	10,686

## Data Availability

The data presented in this study are available on request from the corresponding author. The data are not publicly available due to possible commercial competition.
